# Delayed perforation after cold snare polypectomy for small colonic polyps in a patient receiving oral corticosteroids

**DOI:** 10.1002/deo2.157

**Published:** 2022-07-20

**Authors:** Hirotoshi Iwano, Takayuki Sato, Yoshifumi Ishii, Suguru Niki, Reiji Sawaya, Susumu Tamakawa, Masataka Yamada

**Affiliations:** ^1^ Department of Gastroenterology and Endoscopy Center Shibetsu City Hospital Hokkaido Japan; ^2^ Endoscopy Center Shibetsu City Hospital Hokkaido Japan; ^3^ Department of Surgery Shibetsu City Hospital Hokkaido Japan; ^4^ Asahi Medical Clinic Hokkaido Japan; ^5^ Department of Pathology Asahikawa Medical Center Hokkaido Japan

**Keywords:** bullous pemphigoid, cold snare polypectomy, colorectal polyps, corticosteroid, perforation

## Abstract

This case report describes a fatal outcome due to delayed perforation after cold snare polypectomy in a patient with bullous pemphigoid receiving oral corticosteroids. Cold snare polypectomy has become the standard treatment for small colorectal polyps because of the procedure's safety and simplicity. In this case, however, corticosteroid therapy and vasculitis may have caused local necrosis and tearing of the intestinal wall. Corticosteroids are widely used, and perforation after cold snare polypectomy is extremely rare. However, some patients on corticosteroid therapy may have special pathologies, such as in this case, and we advise physicians to use appropriate judgment and extreme caution in determining the indication for endoscopic therapy.

## INTRODUCTION

Cold snare polypectomy (CSP) was first reported in 1992 as a safe and effective treatment for small colorectal polyps.^1^ The European Society of Gastrointestinal Endoscopy's latest clinical guideline recommends CSP as the preferred technique for removing diminutive polyps,^2^ and CSP is considered the standard method for resecting small polyps.^3^ Although several cases of perforation after CSP have been reported,^4^, ^5^ no case of delayed perforation has been published in the literature. In this report, we describe a case of delayed perforation after CSP.

## CASE REPORT

**FIGURE 1:**
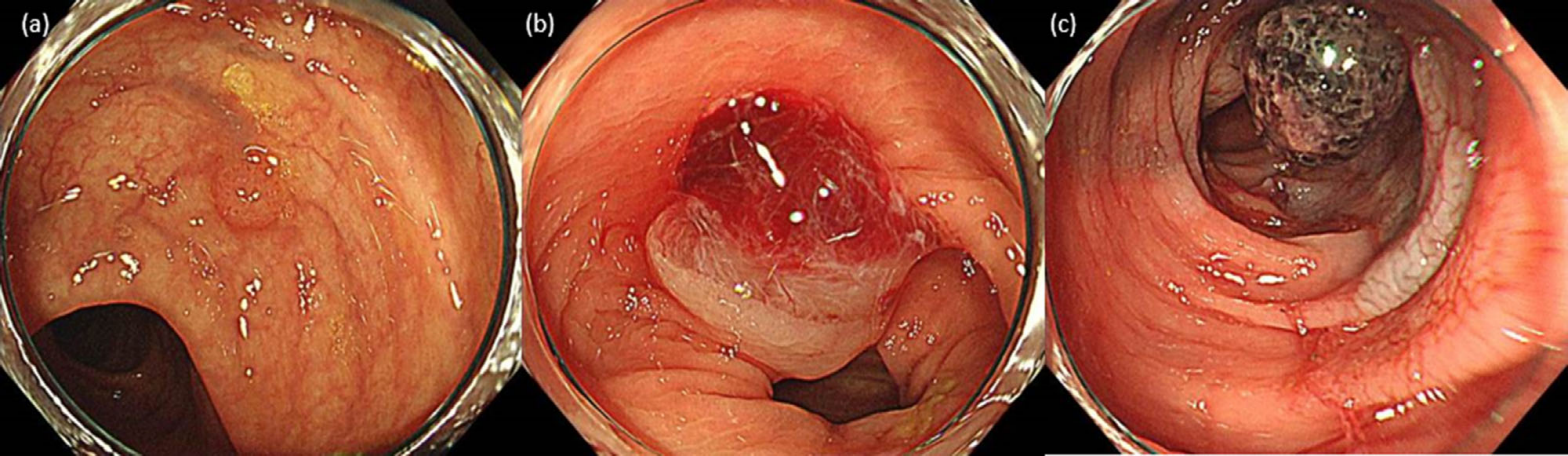
Endoscopy image before perforation. (a) Small polyp (3 mm in diameter) in the transverse colon. (b) After cold snare polypectomy. (c) The day after treatment. A clot adhered to the incision site, and submucosal hemorrhage was observed around the site.

A 77‐year‐old man undergoing corticosteroid treatment for bullous pemphigoid (18 mg/day of peroral prednisolone) was referred to our center for further examination for constipation. A screening total colonoscopy was performed, and several small polyps were found. One month later, the patient was hospitalized for fatigue due to worsening chronic kidney disease. He underwent hemodialysis seven times, but maintenance dialysis was not introduced. His renal function improved to prehospitalization levels. Three weeks after the end of hemodialysis, the patient requested a polypectomy, and eight colorectal polyps were resected by CSP (Figure 1a,b). The polyp shown in Figure 1 is the site of delayed perforation. Three cut sites were closed with clips to prevent bleeding, but clipping was not performed at the site of delayed perforation. At the time of the procedure, 16 months had passed since the start of corticosteroid therapy. After CSP, the patient experienced bloody stools four times that night and the next morning. Instead of gradually becoming old blood, hematochezia was observed in the morning. The attending physician diagnosed the problem as persistent or intermittent bleeding and decided to perform re‐colonoscopy. Blood was observed from the rectum to the cecum, with clots adhering to most of the incision surfaces. In addition, submucosal bleeding was observed around several post‐CSP sites (Figure 1c). No active bleeding was observed. Clips were added to the sites where bleeding was suspected, but not to the site with delayed perforation.

**FIGURE 2:**
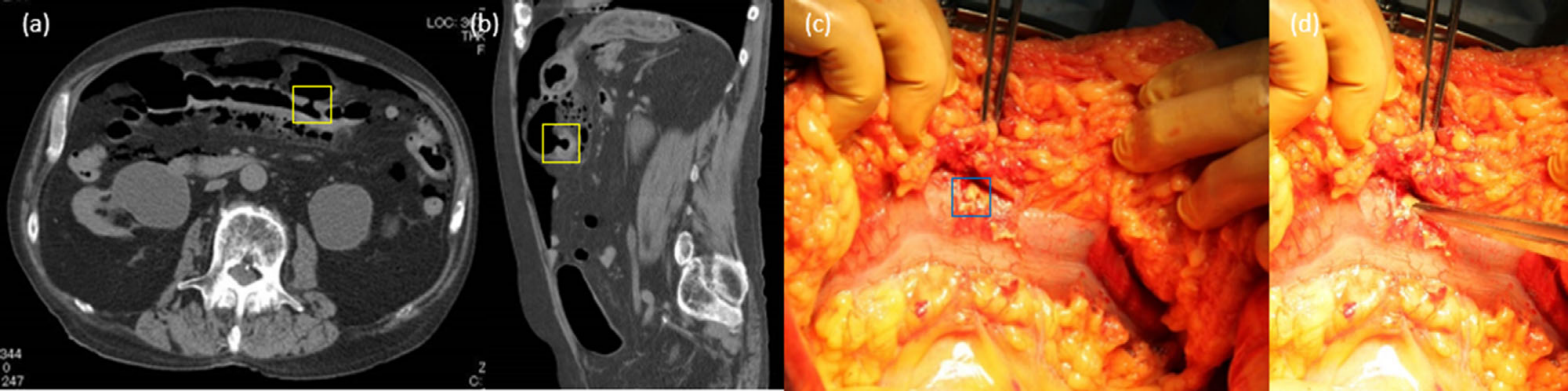
Computed tomography showed free air at the transverse colon perforation site. (a) Axial image. (b) Sagittal image. (c) Intraoperative photograph. The blue rectangle indicates the perforation site. (d) Tweezers are pointed at the perforation site.

Six days after CSP, the patient complained of mild pericardial pain. Two days later, he complained of left upper abdominal pain. Although neither peritoneal irritation nor abdominal guarding was found, free air was present on the X‐ray film, and the computed tomography results revealed a perforated area in the transverse colon (Figure 2a,b). An emergency laparotomy was performed, and a 5‐mm perforation was confirmed (Figure 2c,d). CSP was performed on a 3‐mm polyp in the same area, which was not clipped. Abscesses were found on the dorsal side of the stomach, anterior pancreas, and mesentery of the transverse colon. A partial colectomy that included the abscess was carried out. Two days after the colectomy, water intake was started. Three days later, sinus tachycardia was followed by slow ventricular tachycardia, and shortly after, the heart rate rapidly decreased. Cardiopulmonary resuscitation was performed immediately by the medical staff, but the patient died. His family refused an autopsy. The hospital's investigative committee concluded that the cause of death was fatal arrhythmia caused by severe invasion requiring surgery due to delayed perforation and special patient pathology.

**FIGURE 3:**
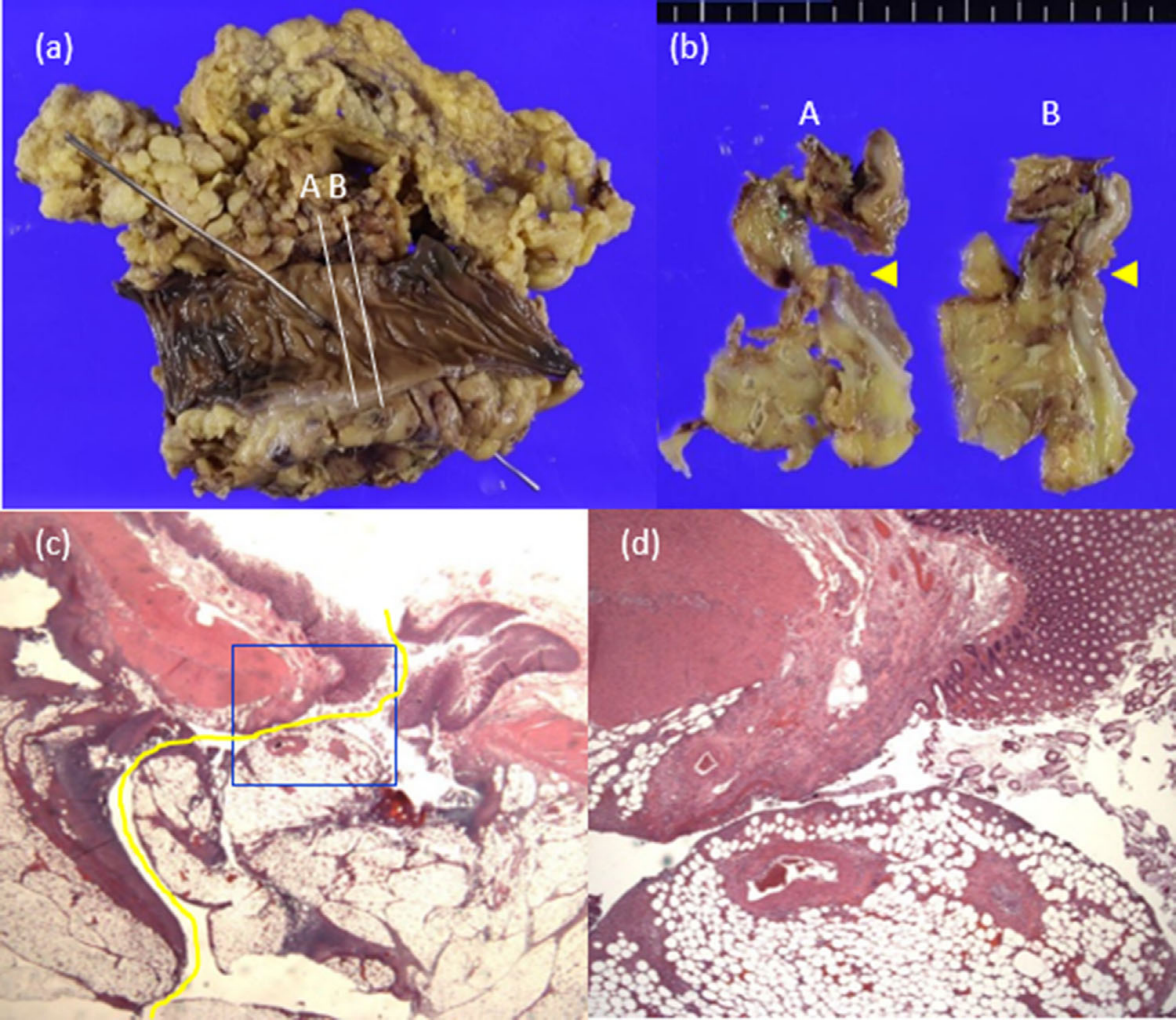
Colectomy specimen. (a) Resected specimen. (b) Cut‐out specimen. The yellow arrowheads indicate the perforation sites. (c) Hematoxylin‐eosin at 5×. The yellow line indicates the perforation site. (d) Necrosis of the residual submucosa, tearing of the muscularis propria and submucosa, and mucosal desquamation were observed (20×).

**FIGURE 4:**
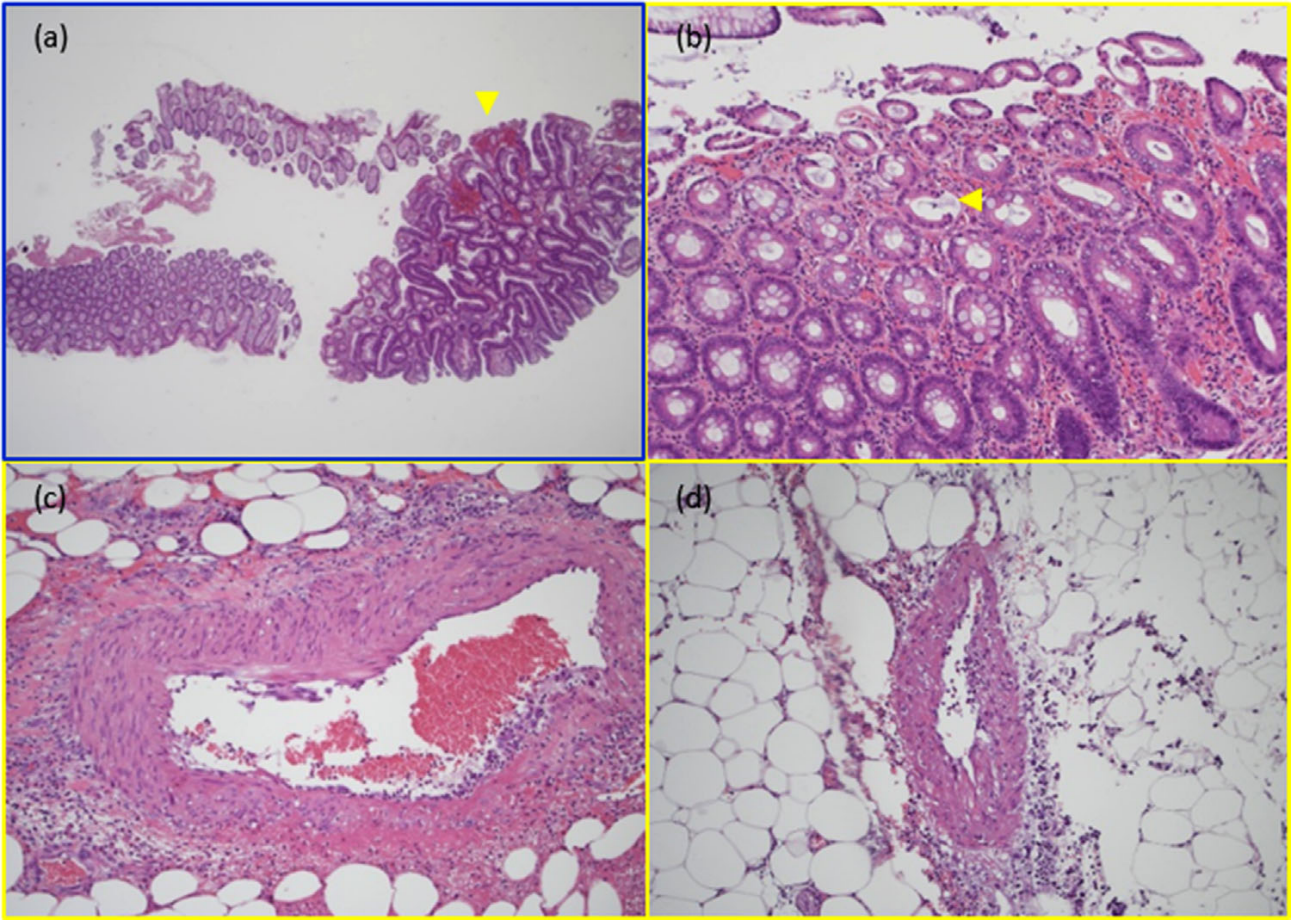
(a) The blue rectangle indicates the histological examination of the cold snare polypectomy specimen, and (b–d) the three yellow rectangles indicate the histological examinations of the resected transverse colon. (a) A small amount of muscularis mucosae was contained in the resected polyp, but muscularis propria was not. The yellow arrowhead indicates focal microbleeding (20×). (b) Atrophy of the mucosa and epithelial desquamation indicating ischemia were observed in the mucosa at the perforation site. The yellow arrowhead indicates epithelial desquamation (100×). (c) Inflammatory cell infiltrations around the middle artery were observed in the subserosa of the perforation site (100×). (d) Vasculitis was also observed at 65 mm from the perforation site (40×).

The CSP and colectomy samples were carefully investigated, and several characteristic findings were identified. The colectomy specimen showed necrosis of the residual submucosa, tearing of the submucosa and muscularis propria, and mucosal desquamation at the perforation site (Figure 3a–d). The CSP specimens did not contain muscularis propria. Localized microhemorrhages were also observed in the mucosa (Figure 4a). Other resected polyps had similar findings. Mucosal atrophy and epithelial desquamation suggestive of ischemia were observed at the perforation site of the resected colon specimen (Figure 4b). Inflammatory cell infiltrations around the middle artery were also observed in the subserosa of the perforation site (Figure 4c). In addition, vasculitis was observed 65 mm away from the perforation site (Figure 4d).

## DISCUSSION

CSP carries little risk of perforation, and resection depth after CSP is more superficial than after hot snare polypectomy.^6^, ^7^ In this case, the CSP specimen of the delayed perforation site (Figure 4a) contained only a small amount of muscularis mucosae and no muscularis propria. Residual tissue loss due to cauterization was not possible, and we believe that the perforation was not caused by the CSP technique. Therefore, we presume that corticosteroid therapy was associated with the perforation.

The mechanism by which corticosteroids cause gastrointestinal bleeding and perforation has not been fully established. In general, corticosteroids can induce ulceration and immunocompromised states because of protein catabolism and anti‐inflammatory properties. The retardation of epithelization, angiogenesis, and wound shrinkage can cause delayed wound healing and intestinal wall weakness and thinning. Furthermore, the analgesic properties of corticosteroids may mask the symptoms of complications, resulting in delayed diagnosis.

According to a meta‐analysis, corticosteroids increase the risk of bleeding or perforation by 40%.^8^ Other reports describe perforations that might have been caused by tissue ischemia, such as vasculitis.^9^, ^10^ A case of unusual jejunal perforation in a patient undergoing corticosteroid treatment described marked hemorrhage and necrotic changes in the mucosa and hemorrhage and inflammatory exudates in the serosa at the perforation site. In that case, pathologic examination of the intestinal vessels showed mesenteric small‐vessel vasculitis and vascular thrombosis with perivascular inflammatory cell infiltration. The authors reasoned that the perforation was due to intestinal ischemic changes.^10^ Another case report of CSP perforation in a corticosteroid‐treated patient described no significant histological findings, such as thinning or weakness of the intestinal wall, but they found focal microbleeding in the polyp.^4^

It is known that subcutaneous bleeding caused by capillary vessel weakness is often seen in Cushing's syndrome, a chronic glucocorticoid surplus. Whether endogenous or exogenous, the capillary fragility caused by corticosteroids predisposes patients to bleed, and even minor tissue damage, such as that from CSP, can cause unexpected bleeding.

In Figure 1c, a large hematoma appears after excision, which is considered to be one of the causes of perforation. We also found focal microbleeding not only in the polyp at the perforation site but also in other polyps, similar to the aforementioned report.^4^ These findings may indicate capillary fragility.

It is also important to note that when the endoscopists resected the polyps, they felt that the polyps were resected more easily than usual, possibly indicating weakness of the intestinal wall. However, other reports describe perforations that might have been caused by tissue ischemia, such as vasculitis.^9^, ^10^ In our case, vasculitis was found in the resected specimen, although no thinning of or abscesses within the intestinal wall existed. The presence of vasculitis might have been one of the causes of the perforation. It is not common for bullous pemphigoid to be associated with vasculitis, and it is likely that a different pathology existed for the vasculitis in this case. Furthermore, the symptoms caused by vasculitis may have been masked during corticosteroid treatment. Since the vasculitis was not pathologically diagnosed until after death and an autopsy was refused, the cause could not be determined.

Although the association between chronic kidney disease and vasculitis is also unknown, the cause of chronic kidney disease was diagnosed in our case as follows. The patient needed to self‐urinate due to dysuria caused by prostatic hypertrophy, but he had repeatedly self‐interrupted self‐urination for seven years and had been hospitalized three times for acute renal failure. His physician diagnosed a gradual deterioration of renal function due to noncompliance. For these reasons, we conclude that the delayed perforation was caused by corticosteroid therapy and vasculitis. These factors may have had baneful influences on the intestinal wall, including unexpected massive bleeding, delayed wound healing, focal ischemic change, and focal necrosis. In this case, a clean colon should not have been the aim, and diminutive polyps should not have been removed. Only the polyps with the potential to be prognostic determinants should have been treated.

We experienced a case of delayed perforation from CSP in a corticosteroid‐treated patient with vascular fragility and the presence of vasculitis. It is possible that some patients on corticosteroid therapy may have a special pathology, such as that of this case, sigmoid colon perforation,^9^ or jejunal perforation,^10^ but it is difficult to confirm in advance whether all patients undergoing colonoscopy have a possible special pathology. To avoid such rare adverse events, clinicians should be wary of patients undergoing corticosteroid therapy. We recommend that the indication for treatment should be evaluated individually before removing polyps.

## CONFLICT OF INTEREST

The authors declare no conflicts of interest.

## FUNDING INFORMATION

None.
